# Impact of guideline definitions on right ventricular diameter in echocardiography: an automated analysis in controls and patients with pulmonary hypertension

**DOI:** 10.1186/s44156-026-00118-2

**Published:** 2026-06-08

**Authors:** Frida N. Hermansson, Bettia E. Celestin, Shadi P. Bagherzadeh, Gracia Fahed, Everton J. Santana, Neha Mantri, Roham T. Zamanian, Alison L. Marsden, Seraina A. Dual, Michael Salerno, Francois Haddad

**Affiliations:** 1 Stanford Cardiovascular Institute, Stanford University, Stanford, CA USA; 2 Division of Pathology, Department of Medicine, Stanford University, Stanford, CA USA; 3 Division of Cardiovascular Medicine, Department of Medicine, Stanford University, Stanford, CA USA; 4 Research Unit Hypertension and Cardiovascular Epidemiology, Department of Cardiovascular Sciences, University of Leuven, Leuven, Belgium; 5 Vera Moulton Wall Center for Pulmonary Disease, Stanford, CA USA; 6 Division of Pulmonary, Allergy & Critical Care Medicine, Stanford University, Stanford, CA USA; 7 Division of Pediatric Cardiology, Department of Pediatrics, Stanford University, Stanford, CA USA; 8 Department of Bioengineering, Stanford University, Stanford, CA USA; 9 Institute for Computational and Mathematical Engineering, Stanford University, Stanford, CA USA; 10 Department of Biomedical Engineering and Health Systems, KTH Royal Institute of Technology, Stockholm, Sweden; 11 Department of Medicine and Radiology, University of California, San Francisco, San Francisco, CA USA

**Keywords:** Right heart, Right ventricle, Transverse diameters, Guidelines, Engineering, Centerline method, Pulmonary hypertension, Reference values, Echocardiography

## Abstract

**Background:**

To improve standardization, the 2025 American Association of Echocardiography (ASE) guidelines revised the definitions of right ventricular (RV) basal and mid diameters, revising the 2010 ASE and European Society of Cardiology (ESC) definitions.

**Objectives:**

(1) To quantify differences in right ventricular (RV) diameter measurements between the 2010 and 2025 ASE guidelines, (2) to determine its impact on the diagnosis of RV enlargement (RVE), and (3) to compare these findings with a novel automated non-linear centerline-based approach.

**Methods:**

We analyzed 208 healthy volunteers and 221 patients with pulmonary arterial hypertension (PAH). Using custom cardiac contour analysis (C2A) software, RV diameters were measured across three definitions. The 2010 ASE/ESC defines diameters as the maximal dimension in the basal and mid segments. In contrast, the 2025 ASE defines the basal diameter just below the tricuspid valve and the mid diameter at ~ 50% of RV inflow, both parallel to the annulus. The C2A method uses a non-linear RV centerline as a reference to standardize measurement locations: the mid-point is taken at 50% along the centerline, and the basal diameter is defined as the maximum perpendicular distance within the proximal third of the RV. We quantified nominal and relative definition differences and compared sex-specific RVE prevalence in healthy controls. Diagnostic performance (ROC) and outcome prediction (Cox models) are presented.

**Results:**

The median age was 63 years in healthy volunteers (52% male) and 48 years in patients with PAH (22% male), with a median pulmonary vascular resistance index (PVRI) of 22 Wood units·m². Compared to ASE/ESC 2010 definitions, ASE 2025 diameters were 15% (basal) and 20% (mid) lower. Using the 2025 definition in healthy controls, basal RVE was observed in 1% of females and 22% of males, while mid-RVE was present in 6% of females and 35.8% of males. The 2010 definition showed slightly better discrimination for PAH (AUC 0.937 vs. 0.898, *p* < 0.001). However, the prediction of outcomes was similar between the two definitions, with C-statistics of 0.59 (0.53–0.65) and 0.59 (0.53–0.66), respectively.

**Conclusion:**

Differences in guideline definitions of RV diameters are important to consider when implementing them in clinical practice.

**Supplementary Information:**

The online version contains supplementary material available at 10.1186/s44156-026-00118-2.

## Introduction

The American Society of Echocardiography (ASE) recently published updated guidelines for assessing the right ventricle (RV), with the goal of improving standardization [1]. A key parameter in clinical practice is the RV diameter from the RV-focused 4-chamber view in 2D echocardiography, specifically the basal and mid-cavity dimensions [1–3]. However, variability exists in how these diameters are defined, particularly in terms of measurement location, orientation, and timing or phase within the cardiac cycle [1–3].

The 2010 ASE, supported by the European Society of Cardiology (ESC), guidelines defined the RV basal and mid diameters as the maximal dimensions within the basal and mid thirds of the chamber [2]. In contrast, the 2025 update defines the basal diameter as immediately below the tricuspid annulus and the mid-cavity diameter at approximately 50% of the distance to the apex, both measured parallel to the annulus [1]. The multicenter World Alliance Society of Echocardiography (WASE) study adopted the 2010 guidelines standard with diameters measured at the frame just before tricuspid valve closure [4]. One of the most commonly used method in cardiac magnetic resonance uses an orientation parallel to the mid-RV and LV chord [5]. The wide range of definitions underscores the complexity of measuring transverse diameters in an asymmetric chamber like the right ventricle. In many biomedical engineering studies, transverse diameters are typically defined as measurements taken perpendicular to a non-linear centerline, a standard approach used to analyze complex structures such as the aorta or coronary arteries [6–8]. The transverse method provides a more consistent anatomical reference frame for measurements in irregular geometries than methods that lack a centerline or defined reference.

Given the variability in current definitions, our study aimed to assess the clinical impact of deploying different methods for measuring RV transverse diameters. To this end, we developed a novel, automated cardiac contour analysis (C2A) software that identifies the RV apex, constructs a non-linear centerline, and computes transverse diameters perpendicular to this reference. The software can also derive diameters based on various ASE definitions, enabling direct comparison between methods.

Therefore, the objectives of the current study were: (1) to develop an automated software for centerline-based analysis of RV transverse diameters; (2) to evaluate the measurement variability introduced by different definitions of RV transverse diameters; and (3) to determine which definitions best correlate with RV area, hemodynamics, and clinical outcomes.

## Materials and methods

### Study design and selected cohorts

The study consisted of two cohorts: the first included prospectively recruited healthy volunteers, and the second included patients with pulmonary arterial hypertension (PAH). The study was approved by the Stanford Institutional Review Board under protocols 17,357 and 25,673.

We prospectively recruited 232 healthy adult participants (age > 18 and < 95 years) from the Stanford Ellison Longitudinal Aging Study between 2011 and 2015. Participants completed a detailed medical history questionnaire and underwent functional assessment using the New York Heart Association (NYHA) classification system and the Rose Angina Questionnaire (London School of Hygiene & Tropical Medicine). Exclusion factors were: history of cardiovascular disease, lung disease, cancer (except local skin cancers), diabetes mellitus, hypertension, obesity (body mass index ≥ 30 kg/m²), competitive athletic training, NYHA functional class other than I, or poor echocardiographic image quality. Among the 232 individuals, 24 were excluded for the following reasons: obesity (*n* = 12), athletes (*n* = 3), lung disease (*n* = 1), established cardiovascular disease (*n* = 6), and poor image quality (*n* = 2).

The patients with pulmonary arterial hypertension (PAH) were retrospectively identified using the Vera Moulton Wall Center for Pulmonary Vascular Disease Registry (2001–2020). Eligible patients were > 18 years old with a diagnosis of PAH confirmed by right heart catheterization (RHC), defined by mean pulmonary arterial pressure (mPAP) > 25 mmHg, pulmonary vascular resistance > 3 Wood units (WU), and pulmonary capillary wedge pressure (PCWP) ≤ 15 mmHg at diagnosis [9]. We selected patients who had both transthoracic echocardiography (TTE) and RHC within a 3-week interval to ensure a diagnosis of PH at the time of comparison. Patients with portal pulmonary hypertension or complex congenital heart disease were excluded. We note that the 2022 ESC/ERS guidance lowered thresholds for the definition of PAH (mPAP > 20 mmHg; PVR ≥ 2 WU), and this was not applied for the selection of patients.

Of 424 initially screened patients from the RHC database, 240 met the inclusion criteria. Nineteen were further excluded due to unavailable DICOM images (*n* = 2) or suboptimal image quality (*n* = 17), resulting in 221 patients. The main reasons for exclusions based on image quality were: the presence of modified RV views or the inability to visualize the right ventricle. Clinical characteristics (heart rate, systolic and diastolic blood pressure) were collected from electronic medical records and manually curated. The clinical outcome was defined as all-cause death or lung transplantation within 5 years of the echocardiogram, with data collected as part of the pulmonary hypertension registry.

### Echocardiography

**Fig. 1:**
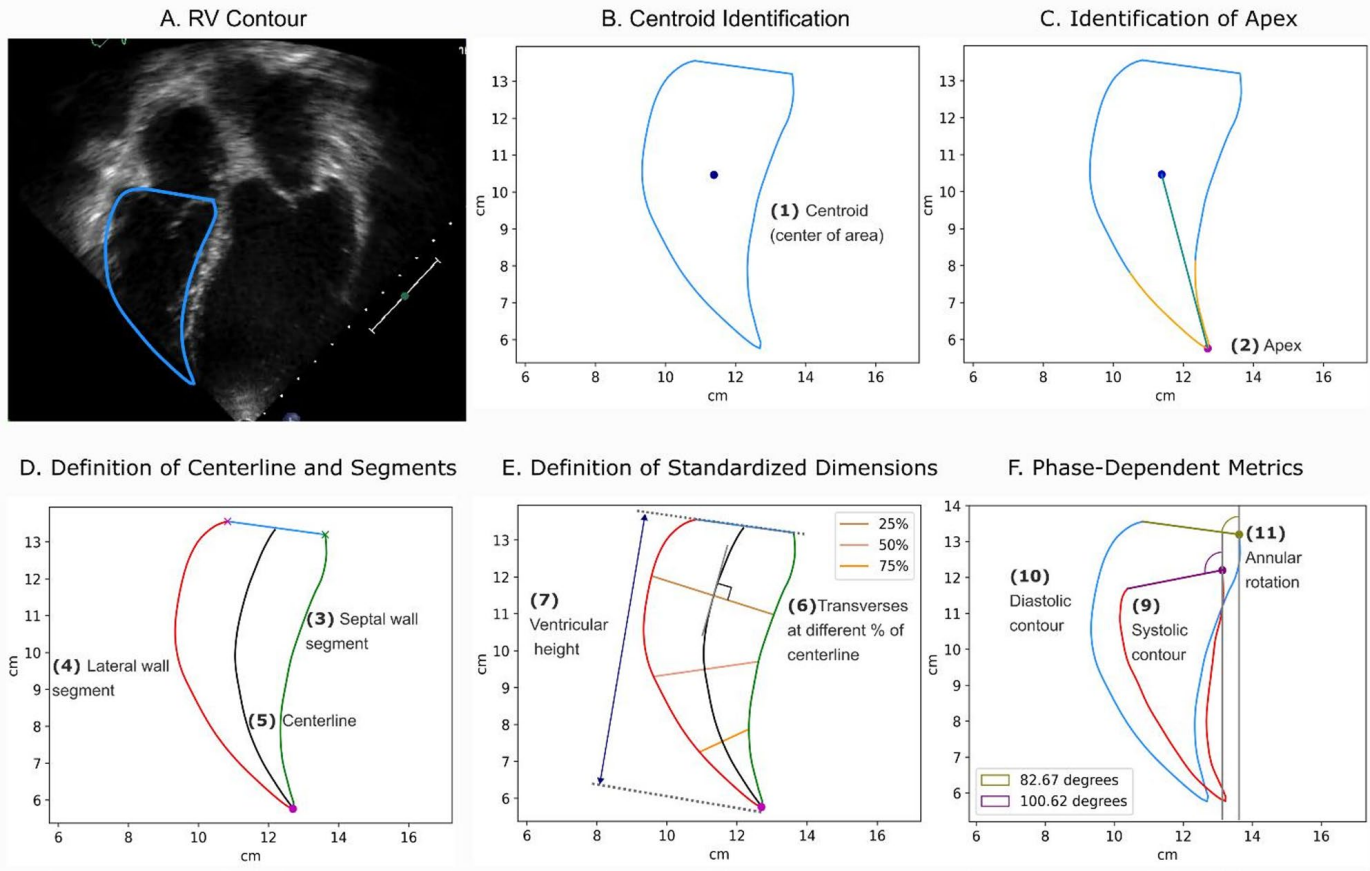
Cardiac contour analysis (C2A) method for the right ventricle. The method starts with an RV contour (**A**) and then identifies the cardiac centroid as the center of the area (**B**). After that, we can locate the apical region and the apex (**C**). This is followed by a derivation of a centerline (**D**), which is further used to derive standardized dimensions of RV height, transverse dimensions, and shape (**E**). Functional measures are further quantified using phase analysis (**F**). Phase-specific cardiac metrics

Two-dimensional echocardiographic images were acquired by trained sonographers or physicians in both the healthy and PAH cohorts using Philips Sonos 7500, iE33, or EPIC 7 ultrasound systems at Stanford Hospital, following ASE guidelines for right heart chamber quantification. [2] In our center, in patients with suspected PH, images were acquired using a dedicated PH protocol since 2006. This protocol emphasized the importance of acquiring dedicated RV views and measuring Doppler signals at the modal frequency. For our analysis, we selected the view most consistent with an RV-focused apical four-chamber view. In the controls, all images were acquired using RV-focused views. RV endocardial contours were traced using the Us2.ai platform by a level 3 reader (FH), and contours were extracted from JSON files (Fig. 1A). End-diastole was defined as the frame with the largest RV cavity, and end-systole as the frame with the smallest cavity. Reproducibility was assessed in all studies as part of an ongoing quality initiative in the Echocardiography Laboratory. RV views were independently selected by FH and BC, and RV areas were measured. Bias and percentile precision were quantified from duplicate analyses, with precision expressed as the standard deviation equivalent (½ × [84th – 16th percentile]) of the scaled (√2) relative difference.

### Automated cardiac contour analysis (C2A) method

From the RV contour, measures of RV size and function were automatically derived using the custom cardiac contour analysis (C2A) software developed by Frida N. Hermansson, MSc. The Python (v3.11.9) script included processing JSON files (downloaded from the Us2.ai system) to extract contour coordinates (*n* = 100 for x and y values), obtain image scaling information of the DICOM file, and conduct subsequent analyses. The geometric analysis of RV contours involved several steps. First, the centroid, representing the center of the area, was identified. (Fig. 1B). Second, the apex was located at the maximal distance from the centroid within a predefined apical region of interest (Fig. 1C). This approach assumes a consistent image orientation and an approximate apex position within the image. Third, the lateral and septal wall segments were identified using the apical and annular reference points (Fig. 1D). Fourth, a non-linear centerline was computed by linking midpoints between lateral and septal wall segments at matching relative positions along the RV (Fig. 1D). Fifth, ventricular lengths, heights, transverse diameters, and geometric ratios were determined with the previously derived centerline as a reference (Fig. 1E). Lastly, phase-dependent ventricular measures, including area changes, were calculated (Fig. 1F). Additional functional metrics were computed within the analysis pipeline; however, these results fall outside the scope of this manuscript and will be reported separately.

### Different guideline-based definitions of transverse RV diameters

**Fig. 2:**
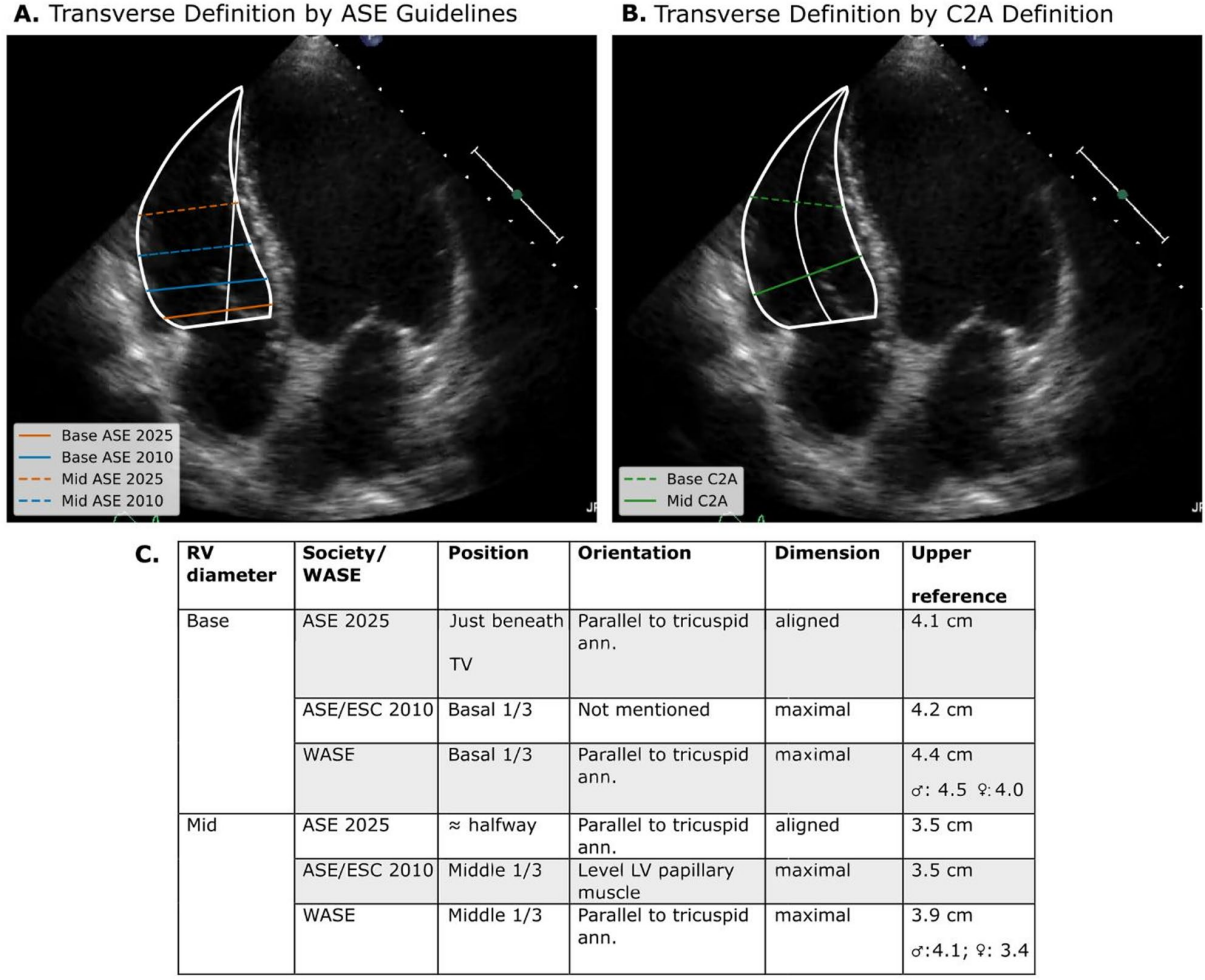
Definitions of right ventricular (RV) transverse diameters. (**A**) Measurement according to the ASE guidelines. (**B**) Illustration of the C2A method. (**C**) Comparative summary of the different transverse diameter definitions. All measurements are recommended on the RV-focused view. The specific cardiac phase (frame) at which measurements should be taken is not specified in the ASE guidelines. The WASE study standardized this phase as the frame immediately before tricuspid valve opening, or, when unavailable, at the peak of the R wave.Abbreviations: ASE, American Association of Echocardiography; TV, Tricuspid valve; ann., annulus; WASE, World Alliance Societies of Echocardiography

**Fig. 3:**
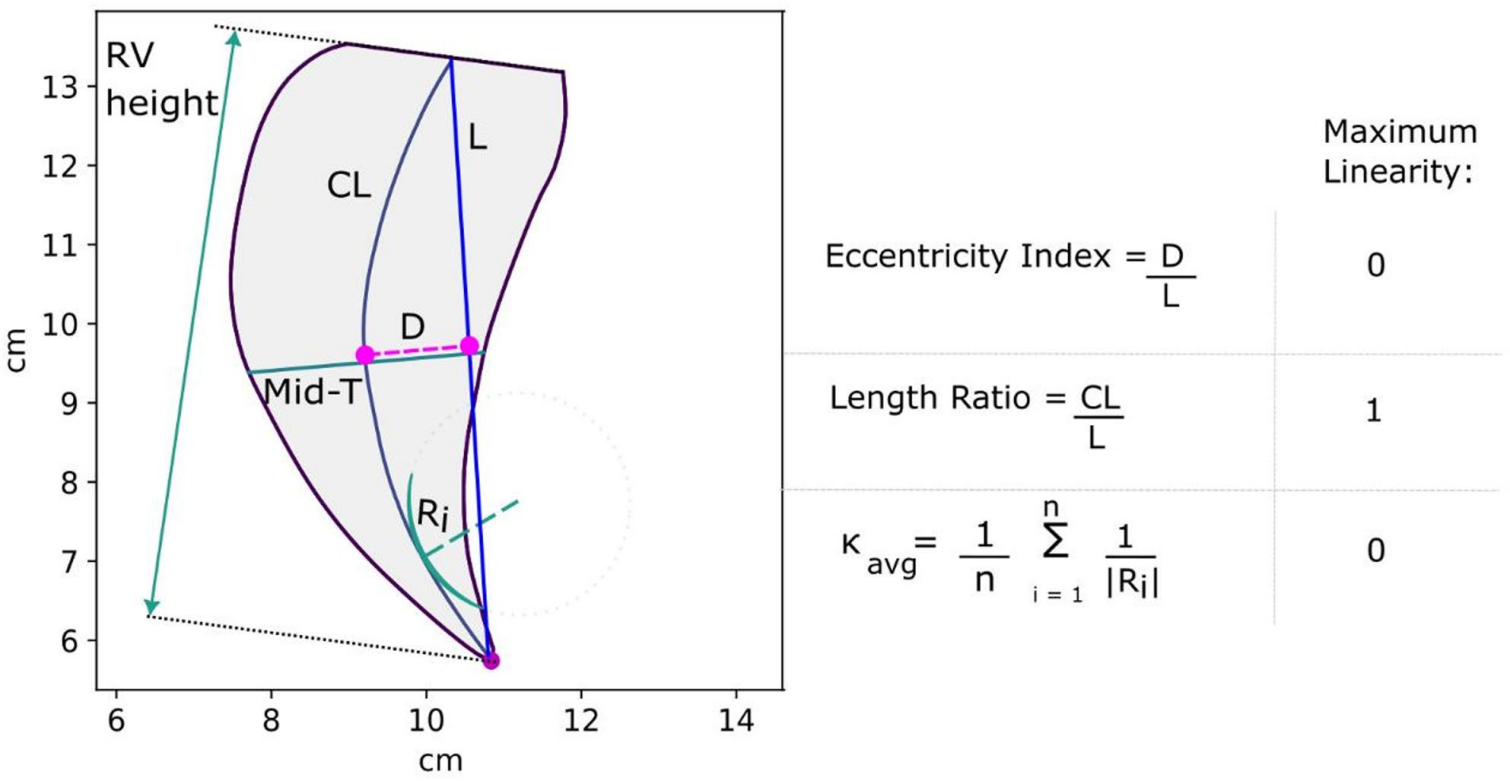
Illustration of RV centerline linearity metrics. The derived centerline (CL) is shown in dark blue, the direct connection between the center of the annulus and the apex (L) in blue, and the direct distance (D) between the midpoint of CL and L is shown in pink. The radius of curvature (R_i_) is indicated for a segment of the centerline. Three metrics were used to quantify linearity: Eccentricity Index (ratio of transverse diameter to length), Length Ratio (centerline to length), and Average Curvature (κ_avg_), calculated as the average of the inverse local radii of curvature along the centerline. The middle column indicates the expected values for a maximally linear centerline

According to ASE/ESC 2010 guidelines [2], the right ventricle (RV) is divided into basal, mid, and apical thirds, with regional boundaries defined along a linear centerline and oriented parallel to the tricuspid annulus (Fig. 2A). According to ASE 2025 guidelines [1], the basal diameter is measured just below the annulus, and the mid-cavity diameter is taken at approximately 50% of the RV inflow tract, both measured parallel to the annular plane (Fig. 2A). A comparative summary of these different transverse diameter definitions is provided in Fig. 2C. In contrast, the C2A method divides RV segments using regional boundaries defined along a non-linear centerline, with transverse diameters oriented perpendicular to this centerline (Fig. 2B). Specifically, the basal diameter is defined as the largest diameter within the basal third of the cavity, and the mid-cavity diameter is measured exactly at 50% along the non-linear centerline, as illustrated in Fig. 2B. This perpendicular orientation aims to better account for RV curvature and asymmetry (see Fig. 3 and the next section).

### Non-linear centerline

In clinical practice, the RV centerline (RV length) is often measured as a straight line, which fails to capture the true curvature of the RV. To address potential challenges in selecting an optimal centerline approach due to the non-linearity of RV centerline curvature, we first assessed whether the assumption of non-linearity was valid. To evaluate the RV centerline curvature, we assessed three quantitative metrics. (Fig. 3): (1) Eccentricity Index (D/L), where values closer to 0 indicate greater linearity; (2) Length Ratio (CL/L), which compares the curved centerline length to the straight-line length (a value of 1 indicates a perfectly linear path); and (3) Average Curvature (κ_avg_), calculated as the average of the inverse local radius of curvature (lower values reflect less curvature). Figure 3 also illustrates the expected values for a perfectly linear centerline.

### Statistical analysis

Analysis was conducted using Python 3.11.5. Numerical values are presented as medians with interquartile range (IQR), while categorical variables are reported as frequency counts (n) and percentages. To compare centerline curvature between healthy controls and patients with PAH, we used the Mann–Whitney U test. Effect size was quantified using Cliff’s delta, which ranges from − 1 to + 1: values near + 1 indicate that all observations in one group are higher than the other, − 1 indicates the reverse, and 0 suggests no consistent difference between groups. To assess paired differences in RV transverse diameter between methods, we used the Wilcoxon signed-rank test, with effect size reported as the median percent paired difference along with the 2.5th to 97.5th percentiles. Spearman correlations and linear regression analysis were also used to analyze relationships between measures.

To evaluate the clinical implications of differences between methods, we conducted several analyses:

1. **Sex-specific thresholds**: We reported the percentage of values exceeding the ASE 2025 single cutoffs, along with the median, 5th, and 95th percentile reference limits. The 5th and 95th reference limits were used in healthy controls due to the modest sample size and the inclusion of healthy recreational cyclists from the San Francisco Bay Area.
2. **Discrimination of PAH**: We assessed the area under the curve (AUC) for PAH classification, comparing basal and mid RV transverse diameter methods using the DeLong method.
3. **Association with pulmonary vascular resistance index** (PVRI): In patients with PAH, we evaluated associations between pulmonary vascular resistance index (PVRI) and RV transverse measures using Spearman correlation, and finally, appropriate scaling was tested by testing body size independence, i.e., absence or minimal associations between indexed measure and scaling parameter.
4. **Clinical outcomes**: For the combined outcome of death or transplantation, we performed Cox proportional hazards modeling, reporting hazard ratios (HRs) and Harrell’s C-statistic to assess prognostic performance.

## Results

### Study population

Among the healthy cohort (*n* = 208), 52% were male (*n* = 109) with a median age of 63 [44,70] years, a majority were of White race (86%), and the median heart rate was 60 [53,66] beats per minute (bpm) (Table 1). In the PAH cohort (*n* = 221), 22% were male, with a median age of 48 [39,58] years, a majority were White race (82%), and the median heart rate was 80 [71,87] bpm. The PAH cohort was a majority of the intermediate to high-risk category, with a REVEAL Lite 2.0 score median of 8 [10]. The C2A method was successfully applied to all tracings except for eight cases, where extreme curvature introduced technical challenges in identifying the mid-transverse plane using certain methods. These cases were therefore excluded from the analysis.

**Table 1:** Healthy population and PAH population characteristics

	Control subjects *N* = 208	PAH *N* = 221
Sex male	52% (109)	22% (48)
Age (years)	63 [44,70]	48 [39,58]
Height (cm)	170 [163,178]	164 [160,170]
Weight (kg)	71 [61,80]	79 [65,89]
BSA (m^2^)	1.84 [1.66,1.99]	1.92 [1.70,2.04]
BMI (kg/m^2^)	24.2 [22.1,26.4]	28.7 [24.0,33.1]
Heart Rate (bpm)	60 [53,66]	80 [71,87]
Diastolic blood pressure (mmHg)	72 [67,79]	72 [64,80]
Systolic blood pressure (mmHg)	119 [109,128]	115 [103,124]
Race		
White	86% (179)	82% (124)
Asian	12% (24)	9.0% (20)
Black	1.4% (3)	3.6% (8)
PVRi (mmHg·min·m^2^/L)	-	22 [14,29]
PVRi severity class		
< 10 (mmHg·min·m^2^/L)	-	9.5% (21/221)
10–20 (mmHg·min·m^2^/L)	-	34% (75/221)
≥ 30 (mmHg·min·m^2^/L)	-	35% (78/221)
≥ 30 (mmHg·min·m^2^/L)	-	21% (47/221)
Reveal lite 2.0 score	-	8 [5,13]
Reveal lite 2.0 class		
≤ 6 Low risk	-	33% (73/220)
7–8 Intermediate risk	-	19% (41/220)
≥ 9 High risk	-	48% (106/220)
LVID (mm)	47 [43,51]	39 [34,43]
LVEF (%)	62 [59,65]	64 [59,70]
RVEDA (cm^2^)	20 [17,22]	34 [29,41]
RVFAC (%)	44 [40,46]	18 [14,24]
RVFWS (%)	28 [26,31]	13 [9,18]

Abbreviations: BMI, body mass index; BSA, body surface area; bpm, beats per minute; CI, Confidence Interval; LVID, left ventricular internal diameter; LVEF, left ventricular ejection fraction; PVRi, pulmonary vascular resistance; RVEDA, right ventricular end-diastolic area; RVFAC, right ventricular fractional area change; RVFWS, right ventricular free wall strain; RVID, right ventricular internal dimension

### C2A method reliability

We evaluated the reliability of the C2A method by (1) visually assessing the placement of the apex, and the level 3 reader (FH) agreed with the visual position of the apex and centerline positions in all but one of the processed cases, where the RV apex was rotated and occupied most of the apical view. All C2A analyses were performed by a single reader (FH) to minimize variability and focus on differences attributable to geometric definitions. Inter-reader variability for RV area was also quantified between two blinded readers, showing a relative bias of − 6.9% and a percentile precision of 7.5% (FH vs. BC).

### Non-linearity of the centerline

Clinical assessment of non-linear curvature requires automated analysis tools and is not feasible through manual evaluation. Therefore, we evaluated the extent of RV centerline non-linearity and its consistency across groups. Non-linearity was quantified using three curvature-based metrics: eccentricity index, length ratio, and average curvature (Table 2). The RV centerline curvature deviated from linearity in both the healthy and PAH cohorts, as indicated by confidence intervals that did not cross 0 or 1, as appropriate. All three metrics demonstrated greater non-linearity in the PAH cohort compared to healthy controls. The difference in centerline curvature between healthy controls and patients with PAH was more pronounced during systole than diastole, with Cliff’s delta ranging from − 0.55 to -0.84 (large differences) in systole and − 0.39 to -0.53 (medium to large differences) in diastole. All differences were statistically significant (*P* < 0.001).

**Table 2:** Assessing centerline curvature using non-linearity indices

RV Centerline parameters	Cardiac phase	Controls *N* = 208^*1*^	PAH *N* = 213^*1*^	Cliff’s delta 95% CI
Eccentricity index	Diastole	0.13 [0.04]	0.16 [0.06]	-0.52 [-0.61, -0.43]
	Systole	0.08 [0.04]	0.15 [0.07]	-0.83 [-0.89, -0.78]
Length ratio	Diastole	1.05 [0.03]	1.08 [0.06]	-0.53 [-0.63, -0.44]
	Systole	1.02 [0.02]	1.07 [0.06]	-0.84 [-0.89, -0.79]
Mean absolute Kappa	Diastole	0.12 [0.03]	0.14 [0.03]	-0.39 [-0.49, -0.29]
	Systole	0.12 [0.04]	0.15 [0.04]	-0.55 [-0.64, -0.46]

Mann–Whitney U tests were performed and yielded p-values < 0.001. Data is presented as median [interquartile range, IQR]. Cliff’s delta effect sizes are reported with 95% confidence intervals (CI). Negative Cliff’s delta values indicate that the PAH group tended to have higher values compared to the control group

Abbreviations: CI, Confidence Interval

### Comparison between transverse diameter methods

**Fig. 4:**
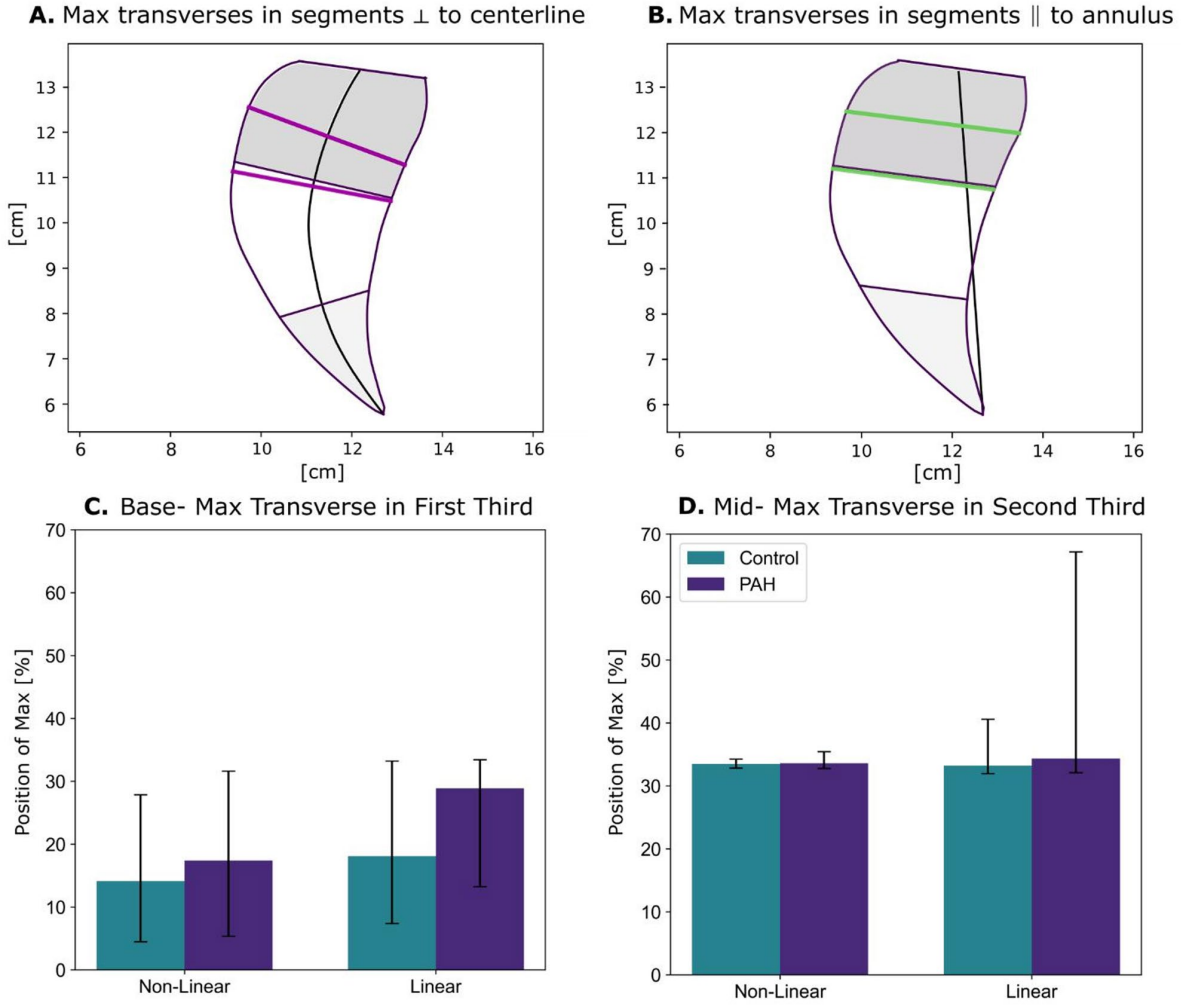
Illustration of two different methods for dividing the right ventricle into thirds. (**A**) Segments are defined perpendicular to a non-linear centerline. (**B**) Segments are defined along a linear centerline and oriented parallel to the tricuspid annular plane. Within these segments, transverse diameters are measured at the basal and mid-levels: the basal diameter is taken from the most proximal segment near the annulus, and the mid-diameter is measured from the middle third of the RV cavity. Bar plots comparing the two methods for basal (**C**) and mid-cavity (**D**) diameters. Bars represent median values, with vertical error bars indicating the 2.5th to 97.5th percentile range

Segmentation may vary based on the choice of centerline (linear or non-linear), the placement of segments, commonly defined in thirds, and the orientation of the transverse cross-sections. This is illustrated in Fig. 4A and B for the non-linear centerline (perpendicular segments) and the linear centerline (parallel to the annulus segments). The position of the maximal diameters is shown in Fig. 4C (RV base) and 3D (RV mid). For the RV base, the maximal diameter was observed at 14.9 [IQR of 6.2] % of the non-linear centerline for controls; 19.1 [IQR of 9.7] % for PAH. The difference between healthy controls and PAH was more pronounced when using the linear method (18.9 [10.7] %for controls; 29.3 [9.1] % for the PAH group). For the RV mid diameter, both the linear (33.1 [1.0] for controls; 34.5 [7.2] for PAH) and non-linear (33.5 [0.5] for controls; 33.6 [0.5] for PAH) methods identified the maximum diameter at approximately 33% of the centerline, with greater variability observed in PAH using the linear method. At the basal level, the differences between control vs. PAH were significant for both methods (Fig. 4).

**Fig. 5:**
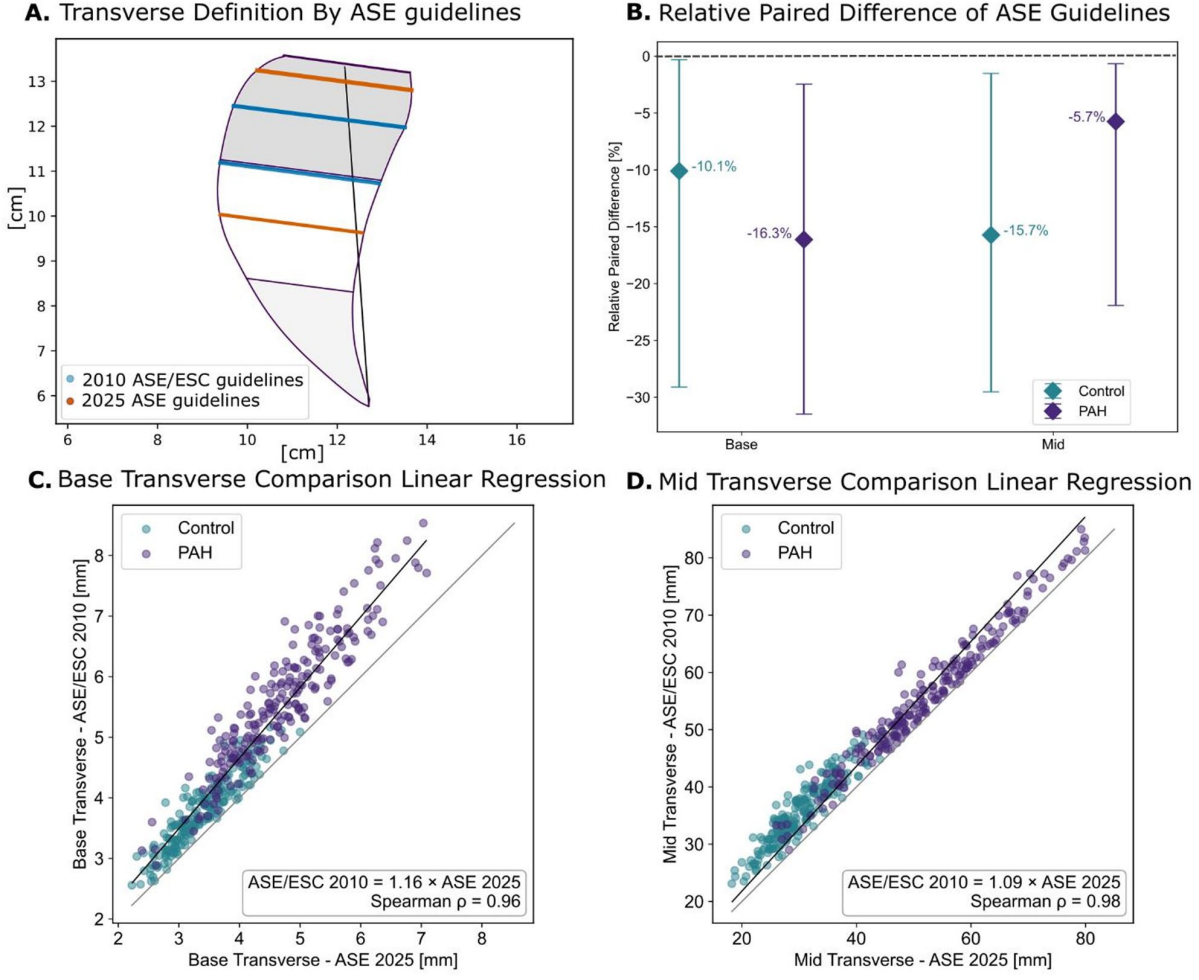
Differences between ASE guideline definitions. Comparison of paired nominal and relative differences in transverse diameters at the base and mid-cavity level of the RV between the new and old ASE guidelines. (**A**) Illustrates how these measurements are defined. (**B**) Shows the paired relative difference (in %) between the metrics. Diamonds represent the median paired difference, with vertical error bars indicating the 2.5th to 97.5th percentile range. (**C**) Shows a linear regression between the two metrics at the base, while (**D**) presents the same analysis for the mid transverse

**Fig. 6:**
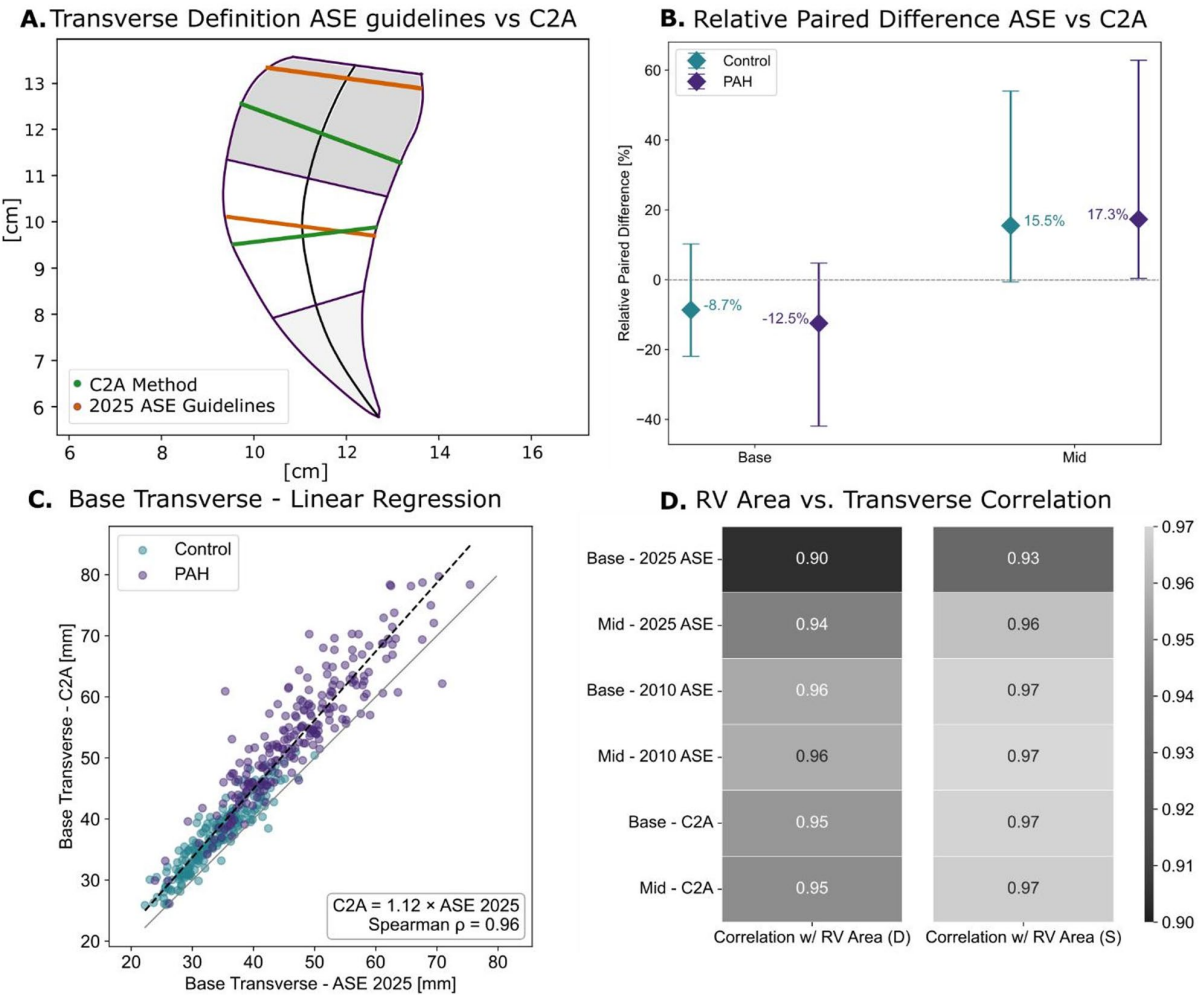
Differences between ASE 2025 guidelines and C2A. (**A**) Shows how measurements from the ASE 2025 guidelines and C2A compare in practice. (**B**) Shows the diastolic paired relative difference (in %) between the metrics, diamonds represent the median paired difference with vertical error bars indicating the 2.5th to 97.5th percentile range. (**C**) Shows a linear regression between the two metrics at the base. (**D**) Shows how the different transverse definitions correlate with the RV areas in each phase

We then compared measurement differences (% paired difference) between the 2010 and 2025 ASE definitions [(2010 standard − 2025 standard)/2010 standard] (Fig. 5) and between non-centerline (ASE) and centerline-based (C2A) methods (Fig. 6). The nominal differences between the two segmentation approaches are shown in Supplemental Fig. 1. While there were very strong associations between ASE definition and Spearman correlation coefficients of 0.96 and 0.98, respectively, paired differences between measures were significant (*P* < 0.001). Systematic differences were more pronounced at the base, with a slope correction factor of 1.16 compared to 1.09 at the mid-level.

Figure 6 highlights the differences between ASE 2025 and C2A reference methods, showing lower values at the base but higher values at the mid-level (*P* < 0.001). These differences appeared systematic, with slope correction factors of approximately 1.12 at the base and 0.84 at the mid-level (*P* < 0.001). Figure 6D illustrates the relationship between the different diameter measurements and RV areas. Illustrative example cases comparing the ASE 2025 and C2A segmentation methods are provided in Supplemental Fig. 2. All diameters demonstrated strong correlations with both end-diastolic and end-systolic areas, with Spearman rho ≥ 0.90. Despite these strong associations, the correlation coefficient for the basal diameter defined by ASE 2025 was lower than that for ASE/ESC 2010 or C2A definitions (*P* < 0.001).

### Clinical implications

#### Prevalence of RV enlargement and reference limits

**Fig. 7:**
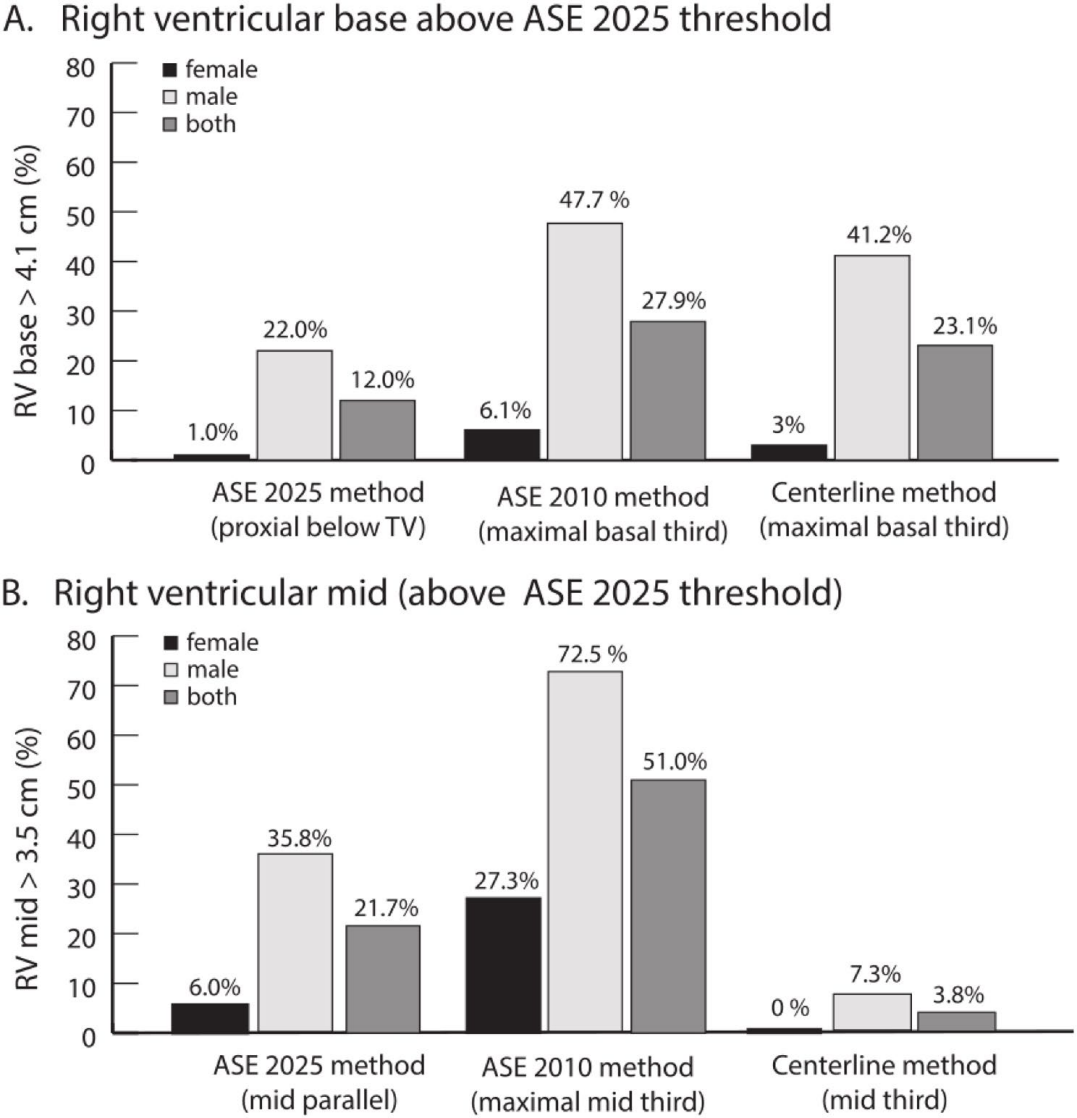
Prevalence of sex-specific right ventricular enlargement applying the ASE 2025 definitions. (**A**) Basal transverse diameter and (**B**) Mid-transverse diameter

When applying the ASE 2025 guideline thresholds to our healthy control group, the prevalence of right ventricular enlargement (RVE) varied not only by RV transverse diameter definition but also notably by sex (Fig. 7). For example, using the ASE 2025 threshold for RV basal diameter, only 1% of females exceeded the cutoff, compared to over 20% of males. These percentages were even higher when using the ASE/ESC 2010 and C2A-based definitions. In contrast, for the RV mid transverse diameter, the centerline-based definition resulted in more consistent percentages of individuals exceeding the threshold across sexes, suggesting improved standardization for reference limits. Reference limits per definition of RV transverse diameters in controls are shown in Table 3, showing that the median difference also translates to the reference limits. Of importance, the reference limits of the ASE equaled or exceeded the median value of the male thresholds.

**Table 3:** Median and limits of reference are measured according to the various definitions

RV diameter	Method	Reference limits ASE WASE		All (*n* = 208)	Male (*n* = 109)	Female (*n* = 99)
Base	ASE 2025	**4.1**		3.3 (2.6–4.3)	3.7 (2.8–4.5)	3.1 (2.5–3.9)
	ASE/ ESC 2010	**4.2**	♂: 4.5*; ♀4.0	3.8 (2.9–4.8)	4.1 (3.1–4.9)	3.4 (2.7–4.2)
	Centerline (max)	**-**	-	3.7 (2.8–4.7)	4.0 (3.1–4.8)	3.4 (2.7–4.1)
Mid	ASE 2025	**3.5**		2.9 (2.2–4.1)	3.3 (2.5–4.2)	2.7(2.1–3.5)
	ASE/ESC 2010	**3.5**	♂: 4.1; ♀3.4	3.5 (2.6–4.5)	3.9 (3.0-4.8)	3.3 (2.5–3.9)
	Centerline (50%)	-	-	2.6 (1.9–3.4)	2.8 (2.1–3.6)	2.4 (1.8–2.9)

Reference values derived from the guidelines for males and females are derived from WASE. Phase is standardized to the largest ventricular dimensions. Since the cohort is smaller, the limits of references are set at the 5th -95th percentiles. * In the 2D WASE paper of Addetia et al. [4] Reference limits in male White males were as high as 4.7 cm based on the mean and 1.96 SD

#### Discrimination of PAH

**Fig. 8:**
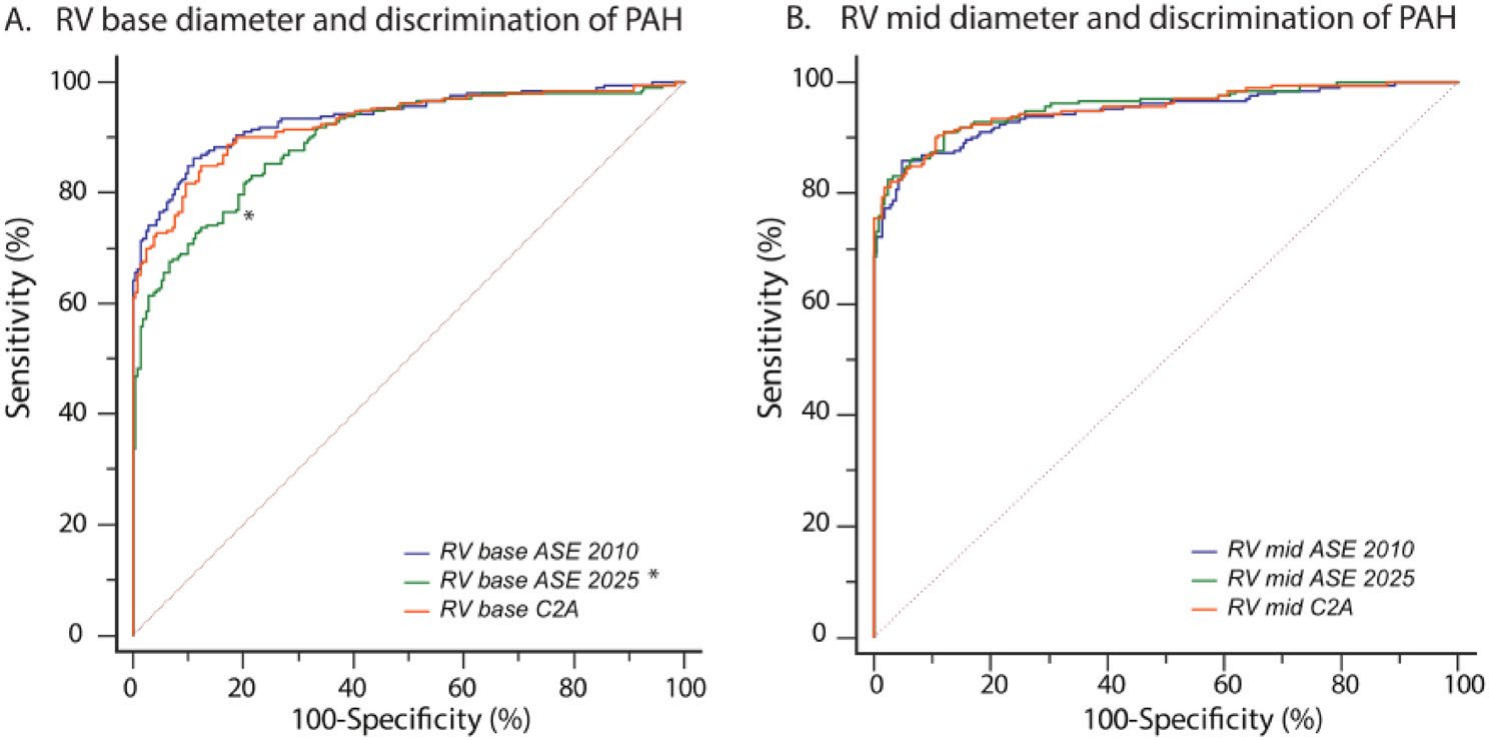
Discrimination of pulmonary arterial hypertension compared to controls using receiver operating curves. Panel (**A**), which focuses on the basal diameter, demonstrates significantly lower discrimination using the ASE 2025 definition (see corresponding value in text). Panel (**B**) shows good discrimination for the mid region, with no statistically significant difference in AUC at α < 0.01

Figure 8 illustrates the impact of different definitions of RV transverse diameters on the discrimination of PAH compared to controls. The AUC presented are based on a case-control study design, which yields more optimistic AUC compared to referral mild cases of PAH. For the RV base, the AUCs for the ASE 2025, ASE/ESC 2010, and C2A definitions were 0.898, 0.937, and 0.927, respectively, with statistically significant differences observed between ASE 2025 and the other definitions (*P* < 0.001). For the RV mid-level diameter, the AUCs for ASE 2025, ASE/ESC 2010, and C2A were 0.956, 0.946, and 0.954, respectively, with no significant differences detected at an alpha level of 0.001.

### Association with hemodynamics and outcome

Prior to analyzing associations with clinical outcomes, we evaluated appropriate scaling metrics for RV transverse diameters in our cohort. As shown in Supplemental Table 1 (healthy controls), indexing RV diameters to height resulted in body size–independent measurements, whereas indexing to body surface area (BSA) introduced a small degree of body size dependence, particularly in males. Despite this, we chose to scale by BSA, as this method has been previously validated and is widely adopted in current clinical guidelines [1]. For hemodynamics, RV diameters showed a weak to moderate association with pulmonary vascular resistance index (PVRI), with Spearman rho values ranging from 0.278 to 0.384 and no statistically significant differences between definitions. In contrast, moderate associations were observed for the RV end-systolic area index (Spearman rho = 0.442, *P* < 0.001) and RV fractional area change (Spearman rho = − 0.515, *P* < 0.001).

Table 4 presents the scaled hazard ratios (HRs) and C-statistics for clinical scores, biomarkers, and selected echocardiographic parameters. The REVEAL Lite 2 score demonstrated the highest hazard ratio, followed by RV areas and NT-proBNP. Overall, RV diameters, regardless of the definition used, showed hazard ratios and C-statistics within a similar clinical range.

**Table 4:** Survival analysis in patients with PAH using scaled hazard ratios

Domain	Variable	HR (per SD Z score)	C-statistic
Score	Reveal-lite ≥ 8	3.10 (1.79–5.35)	0.63 (0.58–0.68)
	Reveal -lite	2.14 (1.61–2.83)	0.69 (0.63–0.75)
	Reveal-Echo	1.68 (1.31–2.15)	0.66 (0.60-0.717)
Biomarker	NT-proBNP (ln)	1.45 (1.12–1.87)	0.61 (0.55–0.68)
RVEDD base index	centerline	1.30 (1.03–1.65)	0.59 (0.53–0.65)
	ASE 2025	1.37 (1.09–1.73)	0.59 (0.53–0.65)
	ASE/ESC 2010	1.34 (1.06–1.68)	0.59 (0.53–0.66)
RVESD mid index	Centerline	1.28 (1.02–1.61)	0.58 (0.52–0.65)
	ASE 2025	1.41 (1.12–1.78)	0.61 (0.55–0.67)
	ASE/ESC 2010	1.35 (1.08–1.70)	0.59 (0.53–0.66)
RVESD index	base (centerline)	1.34 (1.06–1.69)	0.60 (0.53–0.66)
	mid (centerline)	1.30 (1.03–1.64)	0.59 (0.53–0.65_
Areas indexed to BSA	RVEDA	1.50 (1.19–1.87)	0.61 (0.55–0.67)
	RVESA	1.47 (1.16–1.85)	0.61 (0.54–0.67)
Function	RVFAC	0.83 (0.65–1.07)	0.55 (0.49–0.61)

Events applied to 207 patients and include 70 deaths or transplantations at 5 years. Modeling is assumed to be linear (simplification)

## Discussion

Our study demonstrates that guideline definitions of RV transverse diameters are not interchangeable. Using a novel cardiac contour analysis method, we first quantified the curvature of the RV centerline and found greater deviations from normality in patients with PAH. Second, comparison of the 2010 and 2025 ASE definitions showed that maximal diameters, regardless of their location, had the strongest correlations with RV area and most effectively distinguished PAH from controls. Third, in healthy controls, we observed that incorporating sex-specific thresholds is essential to avoid overdiagnosis of RV enlargement. Finally, outcome analyses revealed no meaningful differences in prognostic performance across definitions.

The presence of multiple definitions in the literature reflects ongoing challenges in standardizing RV measurements driven by the complex RV shape [1, 2, 11]. While there is an agreement that the RV-focused view should be used for transverse linear measurements, these measures vary based on the position and orientation of transverse diameters [1, 2, 4]. Moreover, the optimal timing for measurement (e.g., standardized at the maximal area or the frame before tricuspid valve closure) and the definition of the myocardial interface are not consistently reported [11]. The 2010 guidelines measure RV diameters at their maximal dimension in the first and second segments; however, the orientation and cardiac phase are not explicitly specified [2]. In contrast, the 2025 guidelines define the orientation as parallel to the RV annulus, with the basal diameter measured just below the annulus and the mid-diameter measured at the mid-ventricle [1]. The most standardized method used in cardiac MRI follows the approach of Kind et al., where diameters are measured in an orientation parallel to the mid-RV and LV length chords [5, 12]. This method was also adopted by Celestin et al. in their analysis of transverse diameters in echocardiography among patients with PAH [13].

The introduction of these different guideline definitions aimed to address the challenge of standardizing transverse measurements; however, they do not explicitly account for the crescentic curvature of the RV. Unlike the LV, the RV has a non-linear centerline, making visual or manual estimation more challenging and highlighting the need for automated approaches with a clear reference. Using a position parallel to the annual offers an easier “visual” reference line. However, the non-linear centerline, as commonly used in engineering and aortic measures, offers the advantage that it can be applied to measure any segment [7, 14–18].

Consistent with this theoretical rationale, our findings demonstrate that maximal RV diameters, whether derived from the 2010 guidelines or from non-linear centerline methods, are more strongly associated with RV area. In addition, measuring the RV diameter just below the annulus may be challenging due to the curvature of the RV lateral wall. One of the original contributions of our study was the quantification of the impact of differing measurement definitions, revealing a relative median difference of 10–16% in healthy individuals and patients with PAH. These differences carry important implications for detecting RV enlargement with the ASE 2025 definition showing slightly less discrimination for PAH than other definitions.

Reference values in the 2025 ASE guidelines for basal and mid-transverse RV diameters are numerically similar to those in prior recommendations (e.g., 41 mm vs. 42 mm), despite changes in the underlying definitions [1, 2]. The WASE multi-center study, which used the 2010 ASE method, reported average diameters around 44 mm, with the distribution of values showing clear differences by sex and race, reaching up to 47 mm in White males [4]. Consistent with the WASE findings, our study also identified significant sex-based differences in RV measurements, reinforcing the need for refined, sex-specific reference limits. Notably, applying the single threshold values recommended in the recent ASE guidelines resulted in a disproportionately high prevalence of RV enlargement in males, regardless of the measurement definition used. This is particularly relevant, as many clinicians are likely to continue measuring the basal diameter at its maximal dimension in routine practice. Concerning associations with hemodynamics and clinical outcomes, the specific definition of RV diameter showed minimal differences. In contrast, RV areas generally demonstrated stronger associations across both domains.

Another important contribution of this work is the development of an automated analysis workflow. Once the RV contours are delineated, transverse diameters can be automatically extracted, enabling consistent and reproducible measurements. This approach supports the harmonization of definitions and promotes standardization, much like the early development of left ventricular measurement software pioneered by Schnittger and colleagues in the 1980s [19] and current automated workflows in echocardiography [20–22].

### Clinical implications

Our study first demonstrates that not all definitions of RV transverse diameters are equivalent. We show that differences in RV transverse diameters can result solely from geometric definitions. In practice, acquisition and reader variability further contribute to measurement differences, underscoring the need to avoid overinterpreting small longitudinal changes. Our inter-reader analysis further illustrates this point, revealing systematic differences between readers—primarily related to the interface definition of the lateral border—with a relative bias of 6.9% and a percentile precision of 7.5%. Standardization of RV measurements remains a high priority in the field and may explain why the ASE 2025 guidelines adopted a more basal definition for the RV transverse diameter.

A second key implication is that automated contour analysis will likely improve consistency and standardization of RV measurements. The C2A method can be easily integrated into existing echocardiographic software, as it uses routinely acquired contours. Accurate tracing of the RV base and apex, avoidance of apical foreshortening, and inclusion of trabeculations are essential for reliable results. The RV apex is typically located within 1 cm of the LV apex, which can serve as an additional quality control check when tracing RV contours.

### Limitations

The study focused on a cross-sectional cohort, including controls and patients with established PH defined by the pre-2022 criterion (mean pulmonary arterial pressure > 25 mmHg). Therefore, the study does not address how the updated definition may affect diagnostic interpretation in patients with mild PH (mPAP 20–25 mmHg). Our controls also reflect more active individuals in the San Francisco Bay area, which could have led to a higher prevalence of RVE in our healthy cohort. The analysis, however, reveals the importance of not carrying forward the previous definition.

In conclusion, we quantified the differences between existing definitions of RV transverse diameters and demonstrated their implications for assessing RV size, hemodynamics, and clinical outcomes in PAH. Although the 2025 ASE guidelines have suggested changing the RV diameter definition for better standardization, our findings support the use of maximal basal and mid-transverse diameters using anatomically consistent approaches, when feasible, guided by the RV’s non-linear centerline.

## Supplementary Information

Below is the link to the electronic supplementary material.

Supplementary Material 1

## Data Availability

The datasets analyzed in the current study are not publicly available due to patient privacy and institutional data-sharing agreements; however, they are available from the corresponding author upon reasonable request and with appropriate ethical approvals.

## Abbreviations

ASE: American Association of Echocardiography
AUC: Area under the curve
bpm: Beats per minute
BMI: Body mass index
BSA: Body surface area
C2A: Cardiac contour analysis (name)
DICOM: Digital Imaging and Communications in Medicine
ESC: European Society of Cardiology (ESC)
HRs: Hazard ratios
IQR: Interquartile range
κ_avg_: Average Curvature
LV: Left ventricle
LVEF: Left ventricular ejection fraction
LVID: Left ventricular inner diameter
MRI: Magnetic resonance imaging
PAH: Pulmonary arterial hypertension
PH: Pulmonary hypertension
PVRI: Pulmonary vascular resistance index
RHC: Right heart catheterization
ROC: Receiver operating characteristic
RV: Right ventricle
RVE: Right ventricular enlargement
RVEDA: Right ventricular end-diastolic area
RVESA: Right ventricular end-systolic area
RVFAC: Right ventricular fractional area change
RVFWS: Right ventricular free wall strain
TTE: Transthoracic echocardiogram
WASE: World Alliance Societies of Echocardiography
