# Supplementary material for: Impact of guideline definitions on right ventricular diameter in echocardiography: an automated analysis in controls and patients with pulmonary hypertension
Source: Echo Res Pract. 2026 Jun 8;13:21. doi: 10.1186/s44156-026-00118-2 (PMC13244954; doi:10.1186/s44156-026-00118-2)
Supplement: Supplementary file 1 — Supplementary Material 1 [file 44156_2026_118_MOESM1_ESM.docx]

**Supplemental materials** - Impact of Guideline Definitions on Right Ventricular Diameter in Echocardiography: An Automated Analysis in Controls and Patients with Pulmonary Hypertension


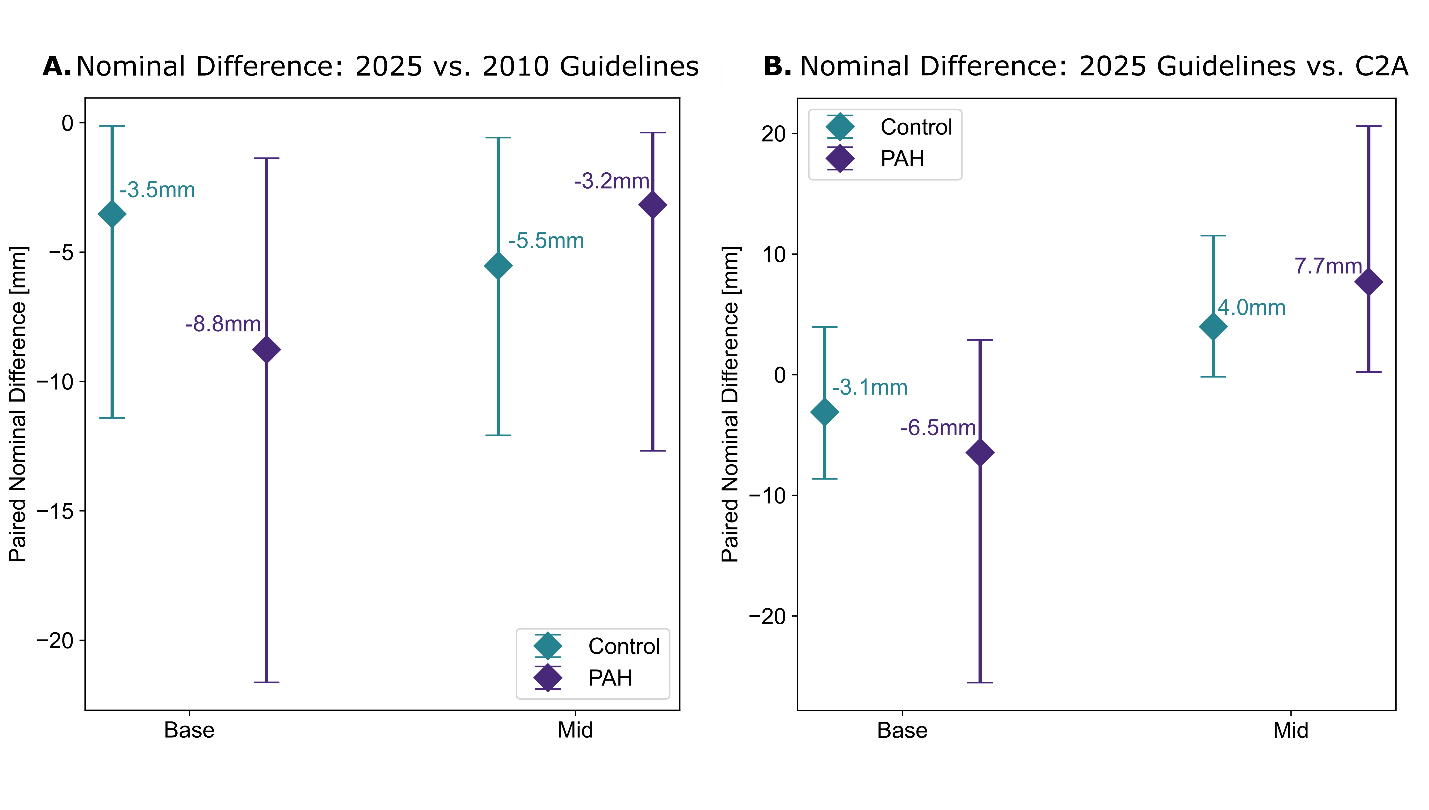


**Supplemental Figure 1. Paired Nominal Differences in Transverse Diameters.**(A) Differences between ASE 2025 and ASE/ESC 2010 guidelines (2010 as reference). (B) Differences between ASE 2025 and C2A measurements (C2A as reference).
Medians are shown with 2.5–97.5 percentile error bars for control and PAH cohorts.


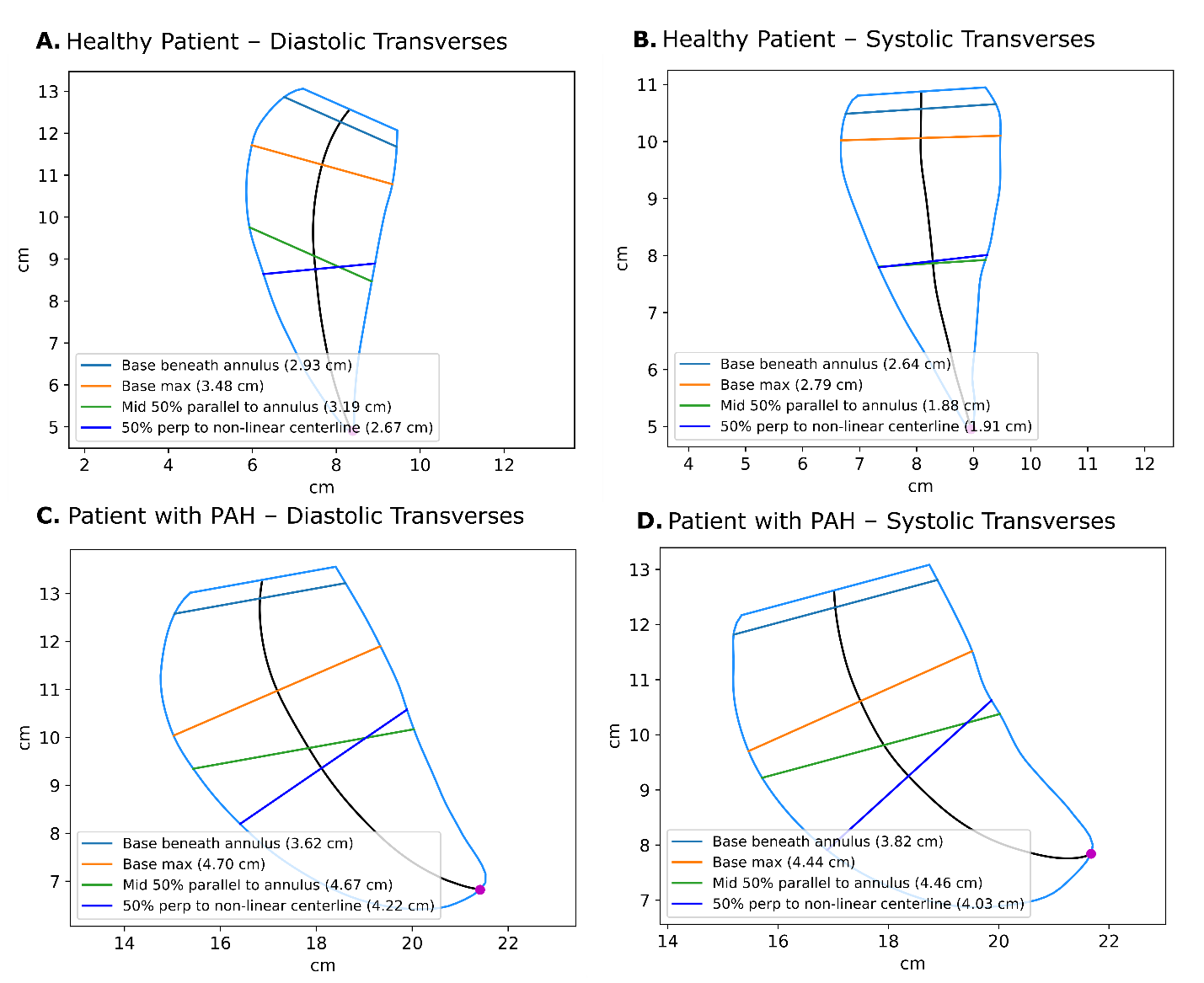


**Supplemental Figure 2.**  **Example cases showing application of ASE 2025 and C2A methods.**(A) and (B): Healthy patient in diastole and systole, respectively. (C) and (D): PAH patient in diastole and systole, respectively.

**Supplemental tables**

**Supplemental Table 1. Dimensional consistency and body size independent scaling**

|  | **Scaling parameter** | **Slope P value (R2 relationship)** | |
| --- | --- | --- | --- |
|  |  | **Male (n=109)** | **Female (n=99)** |
| RV base | **ht** | **0.10** | **0.37** |
|  | BSA | < 0.001 (R2=0.14) | < 0.001 (R2=0.23) |
|  | ht^0.65^ | 0.66 | 0.71 |
|  | BSA^0.30^ | 0.81 | 0.83 |
| RV mid | **ht** | **0.17** | **0.97** |
|  | bsa | 0.001 (R2=0.10) | < 0.0001 (R2=0.16) |
|  | ht^0.73^ | 0.54 | 0.39 |
|  | BSA^0.32^ | 0.86 | 0.71 |
| RVEDA | ht | 0.45 | 0.28 |
|  | **bsa** | **0.006 (R2=0.07)** | **0.52** |
|  | ht^1.3^ | 0.86 | 0.96 |
|  | bsa^0.67^ | 0.27 | 0.43 |

Allometry in the cohort was modeled as a common allometry, adjusting for the sex factor. In bold, selected scaling factors for metrics in association and outcome analysis based on prior literature and body size independence results.

|  |  |
| --- | --- |
|  |  |

**Supplemental Table 2.** Spearman correlations between scaled diastolic metrics and PVRI (n=199)

| **Indexed height metrics** | **C2A** | **ASE 2025** | **ASE/ESC 2010** | **P for difference** |
| --- | --- | --- | --- | --- |
| RV base | 0.364 | 0.307 | 0.334 | ns |
| RV mid | 0.384 | 0.278 | 0.317 | ns |

|  | **Scaling** | **Spearman R** |
| --- | --- | --- |
| RVEDA | ht^1.3^ | 0.275 |
|  | bsa | 0.383 |
| RVESA | ht^1.3^ | 0.357 |
|  | bsa | 0.442 |
| RVFAC | - | -0.515 |

1. Addetia K, Miyoshi T, Citro R, Daimon M, Gutierrez Fajardo P, Kasliwal RR, Kirkpatrick JN, Monaghan MJ, Muraru D, Ogunyankin KO, Park SW, Ronderos RE, Sadeghpour A, Scalia GM, Takeuchi M, Tsang W, Tucay ES, Tude Rodrigues AC, Vivekanandan A, Zhang Y, Schreckenberg M, Mor-Avi V, Asch FM, Lang RM and Investigators W. Two-Dimensional Echocardiographic Right Ventricular Size and Systolic Function Measurements Stratified by Sex, Age, and Ethnicity: Results of the World Alliance of Societies of Echocardiography Study. *J Am Soc Echocardiogr*. 2021;34:1148-1157 e1.
